# FBG-Based Estimation of External Forces Along Flexible Instrument Bodies

**DOI:** 10.3389/frobt.2021.718033

**Published:** 2021-07-30

**Authors:** Omar Al-Ahmad, Mouloud Ourak, Johan Vlekken, Emmanuel Vander Poorten

**Affiliations:** ^1^ Robot Assisted Surgery (RAS), Department of Mechanical Engineering, KU Leuven University, Leuven, Belgium; ^2^ FBGS International NV, Geel, Belgium

**Keywords:** external force estimation, fiber bragg gratings, multi-core fibers, cosserat rod, flexible instruments

## Abstract

A variety of medical treatment and diagnostic procedures rely on flexible instruments such as catheters and endoscopes to navigate through tortuous and soft anatomies like the vasculature. Knowledge of the interaction forces between these flexible instruments and patient anatomy is extremely valuable. This can aid interventionalists in having improved awareness and decision-making abilities, efficient navigation, and increased procedural safety. In many applications, force interactions are inherently distributed. While knowledge of their locations and magnitudes is highly important, retrieving this information from instruments with conventional dimensions is far from trivial. Robust and reliable methods have not yet been found for this purpose. In this work, we present two new approaches to estimate the location, magnitude, and number of external point and distributed forces applied to flexible and elastic instrument bodies. Both methods employ the knowledge of the instrument’s curvature profile. The former is based on piecewise polynomial-based curvature segmentation, whereas the latter on model-based parameter estimation. The proposed methods make use of Cosserat rod theory to model the instrument and provide force estimates at rates over 30 Hz. Experiments on a Nitinol rod embedded with a multi-core fiber, inscribed with fiber Bragg gratings, illustrate the feasibility of the proposed methods with mean force error reaching 7.3% of the maximum applied force, for the point load case. Furthermore, simulations of a rod subjected to two distributed loads with varying magnitudes and locations show a mean force estimation error of 1.6% of the maximum applied force.

## 1 Introduction

Nowadays, flexible instruments and robots are increasingly being used for medical treatment and diagnostic purposes. Thanks to their compliant nature they can navigate through tortuous paths and adjust their shape according to the surrounding environment. Examples of such medical procedures are endovascular aneurysm repair, angioplasty and stenting, thrombolytic therapy, endoscopic gastroscopy, and colonoscopy De Greef et al. (2009); Burgner-Kahrs et al. (2015); Heunis et al. (2018). All these procedures require navigating a flexible instrument through a deformable lumen. For example, in endovascular aneurysm repair a stent catheter is moved through the vaculature. Exerting high forces on soft tissue, for short or extended periods of time, may cause a diversity of complications including perforation, symptomatic intracerebral hemorrhage, bleeding and ischemic complications Balami et al. (2018); Lv et al. (2011); Kavic and Basson (2001). As a consequence, the demand for sensorized instruments relaying crucial real-time information such as: position, shape, and interaction force is ever increasing Shi et al. (2017); Wu et al. (2021); Galloway et al. (2019); Trejos et al. (2010); Haidegger et al. (2009).

The problem of estimating externally applied forces on robotic manipulators is not new. There has been an abundance of works carried out on rigid link robots (Jung et al., 2006; Colome et al., 2013; Daly and Wang, 2014; Hu and Xiong, 2018), and more recently, on soft/continuum robots (Yasin and Simaan, 2020; Sadati et al., 2020; Xu and Simaan, 2010; Gao et al., 2020). Regarding the latter, most of the previous works focused on estimating the contact force at the tip of the manipulator/instrument, rather than along the whole body. For example, Rucker et al. employed an Extended Kalman Filter (EKF) approach and the Cosserat rod model to estimate tip forces using pose measurements and a kinematic-static model of the robot (Rucker and Webster, 2011). Similarly, Hasanzadeh et al*.* made use of pose measurements, and introduced their own quasi-static piecewise circular arc model of the catheter to estimate the tip force (Hasanzadeh and Janabi-Sharifi, 2016). Back et al. use shape information and a simplified Cosserat rod model which can be rapidly solved using an iterative optimization algorithm to estimate forces at the tip of a catheter (Back et al., 2015). Hooshiar et al. estimate tip forces by employing Bezier spline shape approximations and an inverse Cosserat rod model (Hooshiar et al., 2020). Sadati et al. employed force sensor readings at the base of a continuum appendage and the Cosserat rod model for tip load estimation (Sadati et al., 2020). Further examples of previous works on tip force estimation using kinematic models can be found in (Khoshnam et al., 2015; Khan et al., 2017).

As tip force estimation was well understood in recent years, increasing attention was directed towards estimating forces along the length of the flexible instrument body. Qiao et al. made use of curvature measurements using multi-core fibers (MCFs) inscribed with fiber Bragg grating (FBG) sensors, a Cosserat rod model, and linear segmentation of curvature to estimate 2D lateral point forces subjected to an elastic rod body. The feasibility was tested on a Nitinol rod with two point loads (Qiao et al., 2019). In a later work, Qiao et al. employed an extended Kalman filter and pose information in combination with a Cosserat rod model to estimate point loads (Qiao et al., 2021). The approach was validated using two point loads on a thin Nitinol rod. Aloi et al. proposed a method to estimate distributed loads on an elastic rod using a Cosserat rod model and constrained nonlinear optimization. The method was tested using one, two and three point loads in diverse lateral directions along the cross-sectional plane. The paper focused on the feasibility of the proposed method but had a large computational cost and limited use in real-time applications (Aloi and Rucker, 2019). Heunis et al. (Heunis et al., 2019) and Venkiteswaran et al. (Venkiteswaran et al., 2019) modelled a continuum manipulator subjected to multiple external loads using a pseudo rigid body (PRB) model. The location of external loads is assumed to be known a priori. Heunis et al. made use of three-dimensional shape information provided by FBG sensors inside the catheter. Ryu et al. employed shape information, a redundant kinematics model, and an extension of the Cosserat rod model to estimate point loads (Ryu et al., 2020). Lastly, Massari et al. combined machine learning and FBG sensors to develop a tactile sensor capable of estimating the magnitude and location of normal loads exerted on the surface (Massari et al., 2020). The sensor, in theory, can be attached onto the external surface of flexible instruments. Although promising, it is limited with regards to the area of which a force can be exerted upon. In other words, it cannot cover the full circumferential surface of a circular instrument.

In this paper, two new approaches to estimate the *number*, *location*, and *magnitude* of external forces applied onto a flexible and elastic instrument body are proposed. Both approaches rely on Cosserat rod theory as the underlying modelling principle. Additionally, both approaches depart from the availability of curvature measurements. In this work, an MCF inscribed with FBGs is embedded within the instrument’s central working channel to obtain discretized curvature measurements along its length. However, any other method or sensing technology that could also provide similar curvature measurements would suffice. The first estimation approach makes use of piecewise polynomial curvature segmentation, while the second approach employs model-based optimization. The strategies are: 1) initially developed and experimentally tested for two-dimensional point forces applied anywhere along the instrument’s body, and 2) further adapted to estimate both point forces and distributed forces, where a simulation study is provided to prove its feasibility. Note that neither prior knowledge or estimate is required regarding the location of the forces nor an initial identification of whether it is a point force or a distributed force.

According to the above, the contributions of this paper provide the following:

1. force estimation methods that require no prior knowledge of the force interaction, but only knowledge of the two-dimensional curvature profile
2. three new piecewise polynomial-based curvature segmentation algorithms to estimate the number, location, and magnitude of external point forces to high accuracy
3. two new estimation-based approaches that *solely employ curvature measurements* to estimate the number, location, and magnitude of external point and distributed forces to high accuracy, which can be readily extended to include any types of loads
4. static and quasi-static experimental and simulation-based validations of the proposed methods up to three loads applied simultaneously

The rest of this paper is organized as follows: Section 2 briefly introduces the principles of curvature measurement using FBGs and the basic equations of Cosserat rod theory that are used in this work. Section 3 describes the two force estimation approaches in detail. Section 4 covers the experimental setup and the various experimental test configurations. Section 5 presents the results that were obtained. Section 6 will cover the method used for distributed force estimation and a corresponding simulation study. Finally, section 7 provides a conclusion and discussion of the proposed estimation approaches, and comments on future work prospects.

## 2 Sensing and Model Principles

### 2.1 Curvature Sensing

**FIGURE 1:**
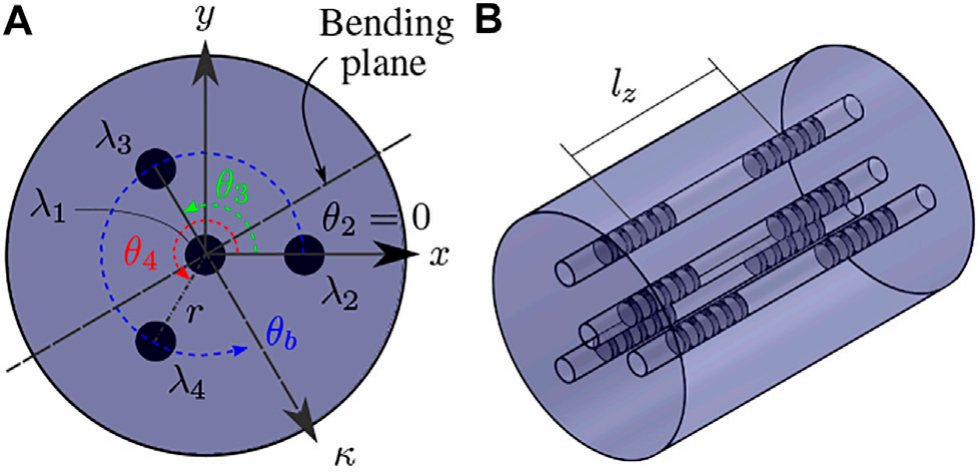
**(A)** cross-sectional view of a four core MCF where $\lambda_{i}$ represents the wavelength in the *i*th core, *r* is the distance between the center of the *i*th core and the fiber’s central axis, and $\theta_{b}$ is the angle of the bending plane with respect to the *x*-axis which intersects the second core, **(B)** isometric view of a segment with four cores and two FBG sets, i.e. four FBGs at a given cross-section, separated by a center-to-center distance $l_{z}$.

FBGs detect variation of strain based on the change of periodicity of a grating, and its refractive index. The Bragg wavelength $\lambda_{B}$ is the wavelength of the light that is reflected back from the grating. The total change in strain can be due to a mechanical strain ϵ, or thermal expansion due to a temperature change ΔT. Considering small temperature shifts, the change in Bragg wavelength can be expressed as:

$$\frac{\lambda_{B}-\lambda_{B_{0}}}{\lambda_{B_{0}}}=\frac{\Delta \lambda_{B}}{\lambda_{B_{0}}}=S_{\varepsilon }\Delta \varepsilon +S_{T}\Delta T,$$ (1)

where $\lambda_{B_{0}}$ is the grating’s unstrained Bragg wavelength, Δϵ is the change in strain with respect to the unstrained state, and $S_{\epsilon }$ and $S_{T}$ are the strain and temperature sensitivity coefficients, respectively. MCFs normally contain a central core that coincides with the fiber’s neutral axis, in addition to a number of symmetrically positioned outer cores. Gratings located within the central core are only sensitive to strain due to axial loading and temperature fluctuations; they are not sensitive to bending as the length of the neutral axis will not alter upon bending. Axial strain is generally assumed to be negligible, and thus the change in wavelength due to temperature change can be simply known by measuring the wavelength shift in the central core ($\text{Δ}\lambda_{B,1}$). For the outer cores, the temperature contribution $S_{T}\text{Δ}T$ is thus known from the central core, and Eq. 1 can be rewritten as:

$$\text{Δ}\epsilon_{i}=\frac{\text{Δ}\lambda_{B,i}}{S_{\epsilon }\lambda_{B_{0},i}}-\frac{\text{Δ}\lambda_{B,1}}{S_{\epsilon }\lambda_{B_{0},1}},$$ (2)

where $\text{Δ}\epsilon_{i}$ is the change in strain in the $i^{th}$ core. The relationship between the strain in each core $\epsilon_{i}$ and the corresponding curvature *κ* can be obtained from the geometry as depicted in Figure 1, and expressed as:

$$\epsilon_{i}=-\kappa r\text{sin}\left(\theta_{b}-\frac{3\pi }{2}-\theta_{i}\right),$$ (3)

where *r* is the distance of the outer cores to the central core assuming a symmetrical configuration (i.e. *r* is equal for all outer cores), and $\theta_{i}$ is the angle of the $i^{th}$. core with respect to the *x*-axis, which intersects the second core. As can be seen from Eq. 3, the two unknowns are curvature *κ* and bend angle $\theta_{b}$, where $\theta_{b}$ is defined as the angle between the *x*-axis and the curvature vector. Hence only two outer core strain measurements are needed to solve for them. However, additional cores can be used to improve the result and reduce errors. A closed-form solution can be obtained for the curvature *κ* and the bend angle $\theta_{b}$ by defining an apparent curvature vector $\kappa_{app}$ Moore and Rogge (2012); Al-Ahmad et al. (2020):

$$\kappa_{app}=-\overset{N}{\underset{i=1}{\sum }}\frac{\varepsilon_{i}}{r}\cos\theta_{i}\hat{i}-\overset{N}{\underset{i=1}{\sum }}\frac{\varepsilon_{i}}{r}\sin\theta_{i}\hat{j},$$ (4)

$$\kappa =\frac{2\;\left|\kappa_{app}\right|}{N},$$ (5)

$$\theta_{b}=∠\kappa_{app},$$ (6)

where $\hat{i}$ and $\hat{j}$ are the unit vectors along the *x*- and *y*-axes respectively, *N* is the number of outer cores, and ∠ represents the vector angle. The bend angle $\theta_{b}$ in Eq. 6 is obtained using the atan2 function. If a MCF contains *n* FBGs located at discrete intervals along its centerline, Eqs. 5 and 6 have to be computed *n* times. This results in a set of curvatures κ[j] and bend angles $\theta_{b}\left[j\right]$ along the fiber’s length with j∈[1,n].

### 2.2 Cosserat Rod Model

**FIGURE 2:**
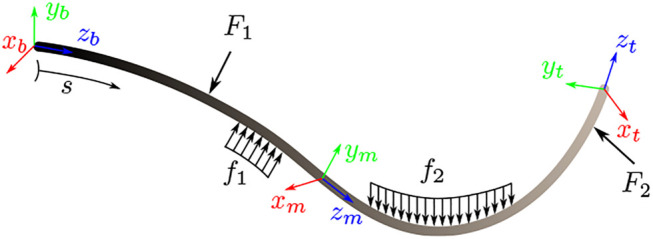
A deformed elastic rod parametrized by arc length *s* subjected to a set of point forces $F_{1}$ and $F_{2}$, and distributed forces $f_{1}$ and $f_{2}$.

The Cosserat rod theory presents a nonlinear mechanics-based model describing elastic rod deformations with regards to internal and external forces. Unlike simpler continuum robot modelling techniques, often relying on constant curvature assumptions, the Cosserat rod model provides accurate and geometrically exact solutions, even for large deflections and curvatures. Figure 2 shows an example of an elastic rod subjected to a variety of external forces. The model is characterized by a set of *equilibrium* equations and a set of *constitutive* equations.

#### 2.2.1 Equilibrium Equations

These equations describe the relationship between external forces, namely distributed forces and distributed moments, and internal reactions, namely internal forces and internal moments. The equilibrium equations which are given as a set of first order differential equations and parametrized by the arc length *s* can be written as Antman (2005):

$$\frac{dn}{ds}=-\hat{u}n-f,$$ (7)

$$\frac{dm}{ds}=-\hat{u}m-\hat{v}n-l,$$ (8)

where n and m are [3×1] internal force and internal moment vectors, respectively, $\hat{u}$ and $\hat{v}$ are the [3×3] angular and linear strain matrices in skew-symmetric form, respectively, and f and l are the [3×1] external distributed force and distributed moment vectors, respectively. Note that all frames and vectors are given with respect to the rod’s material frame (also known as body frame) with their origins being along the rod’s centreline, see Figure 2.

#### 2.2.2 Constitutive Equations

These equations describe the relationship between internal forces and moments with linear and angular strains as:

$$v=K_{v}^{-1}n+v_{0},$$ (9)

$$u=K_{u}^{-1}m+u_{0},$$ (10)

where v and u are the [3×1] linear and angular strain vectors, respectively, $K_{v}^{-1}$ is the [3×3] shear and axial stiffness matrix, $K_{u}^{-1}$ is the [3×3] bending and torsional stiffness matrix, and $v_{0}$ and $u_{0}$ are the [3×1] reference linear and angular strain vectors, respectively. Note that the angular strain u in Eq. 10 corresponds to:

$$u=\left[\begin{array}{c} \kappa \cos\theta_{b}\\ \kappa \sin\theta_{b}\\ 0 \end{array}\right],$$ (11)

where *κ* is the curvature given in Eq. 5 and $\theta_{b}$ is the bend angle given in Eq. 6.

#### 2.2.3 Boundary Conditions

The following assumptions made in this work are outlined first:

• the rod’s axial stiffness is much larger than the rod’s bending stiffness
• there is no, or negligible, linear strain, i.e. $v=v_{0}={\left[0,0,1\right]}^{T}$, such that axial forces are disregarded
• there is no, or negligible torsion, i.e. u[3]=0
• external loads are limited to point forces and distributed forces; there are no point moments nor distributed moments applied on the rod

The *equilibrium* and *constitutive* sets of equations are commonly solved using one of two constraint-based approaches: 1) an approach based on a boundary value problem (BVP), or 2) an approach based on an initial value problem (IVP). The BVP approach would require knowledge of the internal force n and internal moment m at the rod’s “*boundaries*”, i.e. at the base of the rod s=0, and at the tip of the rod s=L, where *L* is the rod’s length. However, the internal forces and moments are commonly unknown at the base. Iterative methods, e.g. shooting methods, are commonly employed to reduce the BVP into a system of solvable IVPs. Alternatively, considering that the rod is rigidly fixed, or cantilevered, at s=0, it is known that the internal force n and internal moment m are both zero at s=L if there is no external force/moment on the tip. Otherwise, the internal force/moment at the tip is equal to the external force/moment. Hence the problem can be reformulated as an IVP starting from s=L with boundary conditions n=0 and m=0 (or $n=F_{ext}$ and $m=M_{ext}$), and solving backwards towards s=0.

Notice that the set of equilibrium equations do not contain terms for externally applied point forces F nor point moments M. The way to include them is to divide the integration process into a smaller set of integrations based on the number and location of the external point loads, and including them as a boundary condition to the internal reactions at that location. For example, suppose that a rod is subjected to a point force $F_{L/2}$ and a point moment $M_{L/3}$ at locations s=L/2 and s=L/3, respectively. The solution can then be found by dividing the problem into three integration sets, starting with n=0 and m=0 at s=L, then $n^{-}=n^{+}+F_{L/2}$ at s=L/2, and finally $m^{-}=m^{+}+M_{L/3}$ at s=L/3. Here, the ^-^ and ^+^ indicates the value of n or m just before the given location (towards s=0) and just after the given location (towards s=L), respectively.

## 3 Body Force Estimation

In this section, two independent force estimation methods are proposed. The first group is based on piecewise polynomial segmentation of measured curvature, while the second group is based on model parameter estimation.

### 3.1 Piecewise Polynomial-Based Segmentation of Curvature

#### 3.1.1 General Approach

**FIGURE 3:**
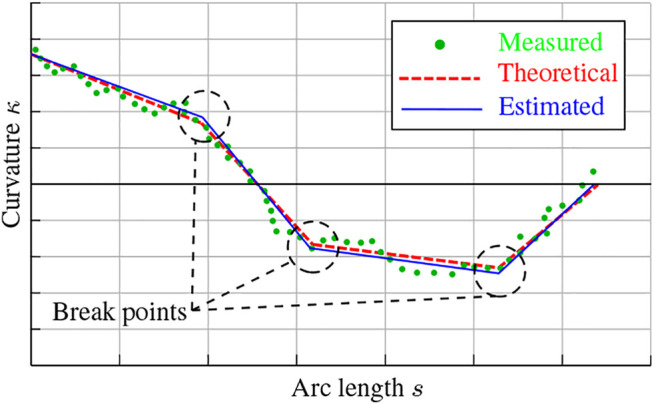
Example plot illustrating the differences between the theoretical, measured, and estimated curvature profiles.

Let us first consider the case where only point forces are applied onto the rod. Looking back at the Cosserat rod’s *constitutive* equations, it can be seen that the internal moment m can be computed in Eq. 10 given knowledge of the angular strain vector u. Notice that the stiffness matrix $K_{u}$ and reference angular strain $u_{0}$ are constants, and that u can be obtained from the measured curvature as seen in Eq. 11. Accordingly, the internal moment computed in Eq. 10 can be numerically derived with respect to the arc length *s*. From the *equilibrium* equations, the derived internal moment can be replaced in Eq. 8 to compute the internal force n (given that l=0 was assumed). Generally, the resulting force profile of n should be continuous. However, when external loads with finite application length are applied, a discontinuity would occur. From this observation, the magnitudes and locations of the external point forces $F_{ext}$ could be found at the break points (see Figure 3), i.e. points of discontinuity, in the internal force profile such that $F_{ext}=n^{-}-n^{+}$. The main drawback with this approach is that the curvature measurements obtained from the MCF tend to be somewhat noisy. Moreover, numerical derivation further amplifies the noise, which jeopardizes the quality of the force estimation.

#### 3.1.2 Polynomials With *n* Degree

Let us now represent the curvature profile of a rod, subjected to a diversity of external forces, as a series of polynomials, each with its own degree *n*. According to the theory of elasticity, for a rod with constant flexural rigidity, the order of the curvature profile resulting from a given force type would be two degrees higher than the force profile itself. For example, a point force has a degree n=−1 and would result in a linear curvature profile, n=1. Similarly, a distributed force with constant magnitude has a degree n=0 and would result in a quadratic curvature profile n=2. Hence, to overcome the noisy curvature measurements problem stated previously, the curvature profile can be segmented into a series of $1^{st}$ order polynomials for point loads, or a series of $1^{st}$ and $2^{nd}$ order polynomials for distributed loads, or a combination of distributed loads and point loads. Note however, that for the final case, segmenting the curvature profile into both linear and quadratic segments is a very challenging task. Especially when there are sparse and discrete curvature measurements available, and when a quadratic segment can be very well approximated by a linear segment with negligible error. Accordingly, to prove the principle behind this approach, the estimation will be limited to point forces only, where an alternative technique will be provided in the second group of estimation methods for distributed forces.

#### 3.1.3 Generic Concept

The concept behind piecewise polynomial-based segmentation of curvature, for the point forces case, is illustrated in Figure 3. Starting from a discrete set of noisy curvature measurements (green dots), a set of linear segments are to be constructed (blue lines) that resemble as close as possible the “*ideal*” theoretical curvature (dashed red lines). Several linear segmentation techniques can be found in literature, see Lovrić et al. (2014) for a review. The most common algorithms include: sliding window, top-down, and bottom-up. Qiao et al. previously employed a top-down linear segmentation approach to estimate one or two point forces applied on a Nitinol rod Qiao et al. (2019). The problem with top-down segmentation approaches, or most conventional linear segmentation approaches for that matter, is that they typically do not consider a penalty on the number of segments constructed. Furthermore, the top-down segmentation algorithm is known for its potential inflexibility in determining the break points Lovrić et al. (2014).

### 3.2 Piecewise Polynomial-Based Segmentation Algorithms

Three alternative linear segmentation algorithms that are specific to the body force estimation problem and provide additional control where possible are proposed. All of the following algorithms start with raw curvature measurements that are merely interpolated to allow for a larger resolution along the arc length.

Algorithm 1 Curvature error thresholding

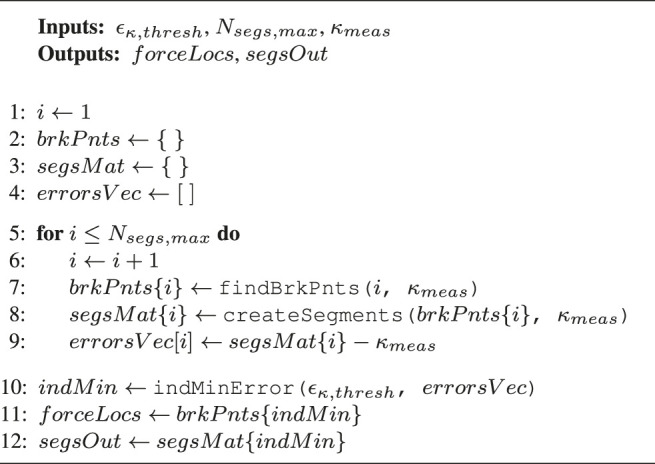

#### 3.2.1 Curvature Error Thresholding

This curvature segmentation algorithm is illustrated in Algorithm 1, and the different properties are described in Table 1. The concept behind this method is that it iteratively varies the number of polynomial segments from $\left[1...N_{segs,max}\right]$, computes the error between the reconstructed segments and the measured curvature, and finally chooses the segments resulting in an error just below a predefined error $\epsilon_{k,thresh}$.

**TABLE 1:** Definition of elements in the different algorithms.

Property	Definition
$\epsilon_{\kappa ,thresh}$	User defined curvature error threshold
$N_{segs,max}$	User defined maximum number of segments to iterate over
$\kappa_{meas}$	Measured curvature from MCF
forceLocs	Estimated force locations
segsOut	A sequence of interconnected linear curvature segments
brkPnts	Structure to store the break points for each iteration; break points are locations of discontinuity in the curvature profile
segsMat	Structure to store the segmented curvature for each iteration
errorsVec	Vector to store the error between $\kappa_{meas}$ and segsMat{i} for each iteration
$F_{min}$	User defined minimum detectable force
$N_{segs}$	User defined a priori estimate on the number of segments
fmin	Minimum estimated force
findBrkPnts (*i*, $\kappa_{meas}$)	Function to compute the optimal *location* of break points based on a desired *number* of break points *i* and a linear computational cost function, see Killick et al. (2012) for details
createSegments (brkPnts{i}, $\kappa_{meas}$)	Function to segment $\kappa_{meas}$ into a series of straight lines based on the given break points brkPnts{i}
indMinError ($\epsilon_{\kappa ,thresh}$, errorsVec)	Function that starts from iteration i=1, checks if the error in errorsVec[i] falls below $\epsilon_{\kappa ,thresh}$; if it does, it returns the number of break points according to the current iteration *i*, otherwise it increments *i* and repeats the process
findMinForce (segsOut)	Function that propagates segsOut through the cosserat rod model and returns the minimum estimated force
mergeBrkPnts (forceLocs)	Function that merges the previously estimated break points based on the location where fmin is found
mergeSegs (segsOut)	Function that merges the previously estimated curvature segments based on the location where fmin is found

#### 3.2.2 Force Magnitude Thresholding

This curvature segmentation algorithm is illustrated in Algorithm 2, and the different properties are described in Table 1. The concept behind this method is that the measured curvature is initially segmented into a user-defined number of segments $N_{segs}$. These segments are then used to compute the forces using the Cosserat rod model. If a force magnitude is lower than a predefined minimum magnitude $F_{min}$ (this happens when the change in slope is very small), the corresponding two segments that caused this force are merged into one segment. The process repeats until there are no more forces below the predefined threshold.

#### 3.2.3 Curvature Slope Change Thresholding

This method relies on the change point detection algorithm used in the *findBrkPnts ()* function, see Killick et al. (2012). This time however, instead of defining the number of segments to find the break points, a minimum slope change $\text{Δ}d\kappa /ds_{min}$ is provided. The algorithm consequently finds the number and location of break points, and the curvature is segmented accordingly.

For the three aforementioned methods, the output curvature segments segsOut are used in the constitutive Eq. 10 to compute the internal moment m. The procedure then continues as explained previously in Section 3, A.1 to compute the magnitudes and locations of the applied external forces. Note that while the illustrated algorithms are restricted to linear curvature segmentation, they can be readily extended to combine both linear and quadratic curvature segmentation.

Algorithm 2 Force magnitude thresholding

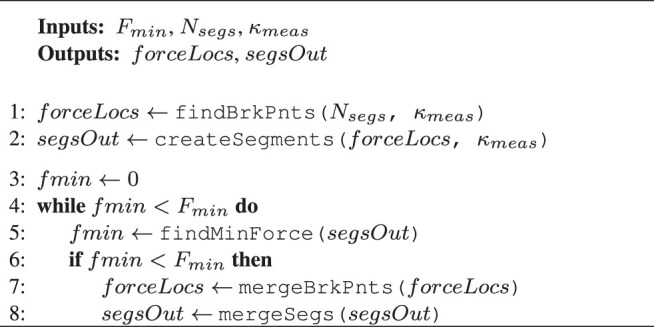

### 3.3 Model-Based Estimation

Here, a predefined number *N* of forces and locations are estimated, where *N* must be larger or equal to the actual number of applied forces $N_{act}$. If it is strictly larger, it will become clear that the magnitudes of those forces additional to $N_{act}$ will be equal to zero, or have a very small value. Let us now consider a function g(χ) that requires an input χ and returns an output κ such that:

$$\chi =\left[\begin{array}{c} F_{x1}\\ F_{y1}\\ L_{1}\\ F_{x2}\\ F_{y2}\\ L_{2}\\ \vdots \\ F_{xN}\\ F_{yN}\\ L_{N} \end{array}\right],\;and\;\kappa =\left[\begin{array}{c} \kappa_{x1}\\ \kappa_{x2}\\ \vdots \\ \kappa_{xM}\\ \kappa_{y1}\\ \kappa_{y2}\\ \vdots \\ \kappa_{yM} \end{array}\right],$$ (12)

where $F_{xi}$ and $F_{yi}$ are the magnitudes of the *x* and *y* components of the $i^{th}$ external point force relative to the rod’s body frame, respectively. $L_{i}$ is the location of the external point force expressed as arc length $s=L_{i}$. $\kappa_{xj}$ and $\kappa_{yj}$ are the magnitudes of the *x* and *y* components of the $j^{th}$ curvature measurement along the arc length, and ***M*** the total number of curvature measurements over the total length. The function g(χ) provides the forward kinematics of the Cosserat rod model, which is basically the inverse of the process described in Section 3, A. The estimation objective is to find χ given measured κ and forward kinematics function g(χ).

#### 3.3.1 Least-Squares Optimization

An optimization algorithm typically finds a set of optimization parameters based on minimizing a desired cost function $\Theta \left(\chi ,\kappa_{meas}\right)$. In this case, the cost function is defined as:

$$\Theta \left(\chi ,\kappa_{meas}\right)=\arg\;\min\left({\left(g\left(\chi \right)-\kappa_{meas}\right)}^{2}\right),$$ (13)

where $\kappa_{meas}$ is the measured curvature vector using the MCF. Hence, the goal is to minimize the error between the measured curvature $\kappa_{meas}$ and the estimated curvature g(χ)=κ, defined by the optimization parameters χ. While there is a myriad of optimization algorithms available in the literature, the interior, bounds-constrained trust region algorithm for nonlinear optimization is employed Le et al. (2017).

#### 3.3.2 Unscented Kalman Filter (UKF)

In a similar way to optimization algorithms, Kalman filters can be used to estimate model parameters. In this case, the model parameters are the point force magnitudes and locations defined in χ. Compared to the linear Kalman filter (KF) or the Extended Kalman filter (EKF), the UKF is advantageous as it can accurately estimate the true mean and covariance of the states, in addition to the posterior mean and covariance to a third order for any nonlinearity. The EKF can only achieve first order accuracy. In addition, the computational complexity of the UKF is in the same order as that of the EKF Wan and Van Der Merwe (2000). Finally, the UKF avoids the need to compute the state and measurement function jacobians, which sometimes have to be computed numerically.

The state transition model and the measurement model of the UKF are described as:

$$x_{k}=x_{k-1}+w_{k},$$ (14)

$$z_{k}=h\left(x_{k}\right)+v_{k},$$ (15)

where $x_{k}$ are the states at time step *k* such that $x_{k}=\chi$, $h\left(x_{k}\right)$ is the measurement function such that $h\left(x_{k}\right)=g\left(x_{k}\right)$, $z_{k}$ is the measurement at time step *k*, and $w_{k}∼N\left(0,\;Q_{k}\right)$ and $v_{k}∼N\left(0,\;R_{k}\right)$ are the process and measurement noise, respectively. $Q_{k}$ and $R_{k}$ are the process and measurment noise covariance matrices at time step *k*, and are found by manual tuning. Note that the states $x_{k}$, i.e. the magnitudes and locations of the point loads, are assumed to evolve through random walk. The remainder of the UKF equations follow the process outlined in Wan and Van Der Merwe (2000), and are omitted here for brevity.

## 4 Experiments

### 4.1 Description of the Experimental Setup

**FIGURE 4:**
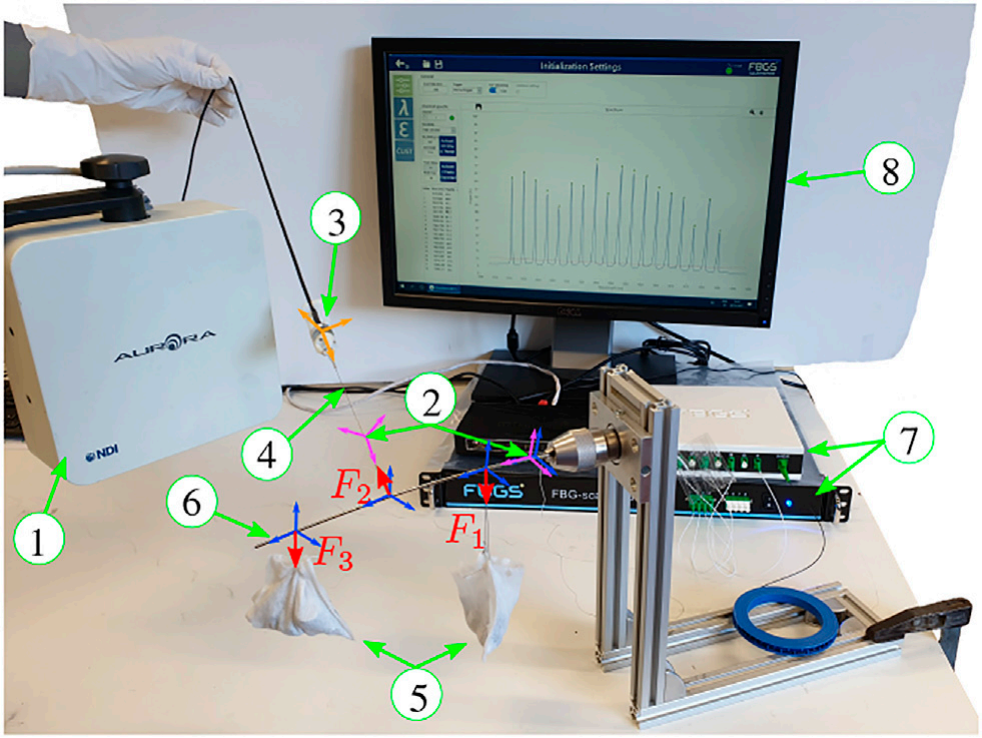
Force estimation experimental setup comprising: ① electromagnetic (EM) field generator, ② electromagnetic tracking (EM) sensors, ③ ATI Nano17 six DoF force sensor, ④ tensioned cable to transmit a force to the Nitinol rod, ⑤ weights suspended by gravity, ⑥ Nitinol rod with an MCF embedded internally, ⑦ optical fanout and interrogator systems, ⑧ monitor to display wavelength spectra. Forces $F_{1}$ and $F_{3}$ are applied by suspending known weights, and force $F_{2}$ is applied along an adjustable direction by pulling on the ATI Nano17 six DoF force sensor which is attached to a cable that encircles the Nitinol rod.

Figure 4 illustrates the force estimation experimental setup. The setup comprises a Nitinol rod Figure 4 ⑥ with an outer diameter of 2.16 mm and an inner diameter of 1.65 mm. The rod is horizontally clamped to an aluminium frame using a chuck with a through hole. A low-friction PTFE tube with outer diameter of 1.60 mm and inner diameter of 0.25 mm is inserted into the lumen of the Nitinol rod. Accordingly, a MCF fiber with an outer diameter of 0.2 mm is inserted into the PTFE tube. At the proximal end, the PTFE tube is rigidly fixed to the Nitinol rod using adhesives. Similarly, the MCF is also rigidly fixed to the PTFE tube using adhesives. The Nitinol rod and PTFE tube combination have a flexural rigidity of 0.048 Nm^2^. The MCF (FBGS International NV, Geel, Belgium) has one central core, three outer cores, and a total of 18 FBG sets with a 10 mm FBG separation distance (see $l_{z}$ in Figure 1) covering a total length of 170 mm. Cable suspended weights Figure 4 ⑤ are used to apply point forces onto the rod in the direction of gravity. An ATI Nano17 six DoF force sensor (ATI Industrial Automation, North Carolina, United States) Figure 4 ③ is attached to a cable, which is wrapped around the Nitinol rod. This construction allows applying a known force (measured by the ATI nano17) in an arbitrary direction on the rod. An NDI electromagnetic tracking (EM) system (Northern Digital, Waterloo, Canada) Figure 4 ① is employed to determine the transformation between the cable tension force measured in the Nano17 sensor frame and the rod’s body frame. One EM tracking sensor Figure 4 ② is thus attached collinearly with the tensioned cable, and another is attached to the base of the rod. An optical fanout and interrogation system (FBGS International, Geel, Belgium) Figure 4 ⑦ is used to measure the wavelength shifts of the FBG sets within the MCF.

### 4.2 Calibration of EM Tracking Sensors With ATI Force Sensor

An EM tracking sensor is rigidly fixed to the cable attached to the ATI force sensor. The total applied force onto the Nitinol rod is equal to the tension force within this cable such that $F_{tot,ATI}=\sqrt{F_{x,ATI}^{2}+F_{y,ATI}^{2}+F_{z,ATI}^{2}}=F_{2}$, where $F_{2}$ is the cable tension force as shown in Figure 4. Note that the weight of the cable is negligible compared to the applied forces. The *z-*axis of the EM tracking sensor frame is aligned with the cable and rigidly fixed in that configuration. To calibrate the direction of $F_{2}$ with respect to the rod’s body frame, two additional EM tracking sensors are used. The first is rigidly attached to the base of the rod at s=0, and the second is attached at the tip of the rod at s=L. The tip of the rod is then moved (by hand) in a spiral path and both EM tracking sensor data and the rod shape are recorded [see Al-Ahmad et al. (2020) for the method used to reconstruct the rod’s shape]. The rigid transformation matrix Trod_*EM*_ between the EM frames and the rod’s shape is found using the rigid Iterative Closest Point (ICP) registration algorithm. Any EM frame can now be transformed to the base EM frame and consequently to the rod’s frame (hence the tip EM sensor is no longer needed and is removed). The cable tension force $F_{2}$, which is aligned with the *z* axis of the cable EM frame is then transformed to the base EM frame and consequently to the rod’s body frame.

### 4.3 Description of Experiments

The following parameters were used in the experiments: $\epsilon_{\kappa ,thresh}=0.05$, $N_{segs,max}=5$, $F_{min}=0.2$ N, $N_{segs}=5$, and $\Delta d\kappa /ds_{\min}=0.45$ m^−1^. Note that $\epsilon_{\kappa ,thresh}$ is a normalized value. Furthermore, N=4, $R_{k}$ = *diag* [*repmat* (0.01 m^−1^, 1, 2*M*)], and $Q_{k}$ = *diag* [*repmat* (0.1 N, 0.1 N, 0.1 m, 1, *N*)] where the function *repmat* (matIn, Nrow, Ncol) returns a matrix that is a repetition of the input matrix matIn for Nrow rows and Ncol columns, and the function *diag* (matIn) returns a diagonal matrix based on the elements of matIn.

Experiments were carried out for one, two and three point forces being applied onto the rod at a given time. Furthermore, for each load configuration, the location of the forces were changed/interchanged along the arc length and their magnitude was varied between 0−5.25 N. The ATI force sensor attached to the cable was also rotated slowly around the rod’s centreline to vary the applied two-dimensional forces quasi-statically. The mean, maximum, and standard deviation of the change in force magnitudes with respect to the ATI nano17 frame were 0.8 N/s, 16.9 N/s and 0.9 N/s respectively. The algorithms were running using c++ code on a Intel(R) Core(TM) i7-8850H, 2.60 GHz dual core processor on an Ubuntu 16.04 operating system.

## 5 Results

In total, there were eight single force, six dual force, and three triple force configurations. For each configuration the ATI force sensor was rotated along the rod’s centreline to vary the lateral *x-* and *y-*components of the force. This resulted in a total of 12,908 recorded forces applied onto the rod for all configurations. Forces with magnitude less than $F_{min}$ (defined earlier in Section 4, C) are discarded as they are considered to be negligible in magnitude and rod deformation. If a method estimates the number of forces to be less than the actual number of forces, then the error for the extra forces are the force magnitudes themselves and the error for their locations are taken as zero. Table 2 shows a summary of the results for the five force estimation methods (three piecewise polynomial-based curvature segmentation methods and two model-based estimation methods). Note that the errors are defined as the absolute value of the difference between ground truth and estimated forces/locations.

**TABLE 2:** Summary of force estimation results illustrating estimated force numbers and errors on magnitudes and locations. E(||⋅||) and $\sigma \left(\left||\epsilon_{\cdot }|\right|\right)$ represent the mean and standard deviation of the absolute value of the error, respectively.

	Method				
**Result**	1.a	1.b	1.c	2.a	2.b
Computation time	∼1−2 ms	∼1−2 ms	∼1−2 ms	∼30 ms	∼8 s
Correct no. of forces	6,089 (47.2%)	10,158 (78.7%)	6,597 (51.1%)	9,845 (76.3%)	8,842 (68.5%)
$E\left(\left||\epsilon_{F_{X}}|\right|\right)$ [N]	0.346 (6.6%)	0.422 (8.0%)	0.372 (7.1%)	0.326 (6.2%)	0.385 (7.3%)
$E\left(\left||\epsilon_{F_{Y}}|\right|\right)$ [N]	0.162 (3.1%)	0.294 (5.6%)	0.149 (2.8%)	0.213 (4.1%)	0.204 (3.9%)
$E\left(\left||\epsilon_{L}|\right|\right)$ [M]	0.01160 (6.8%)	0.00910 (5.4%)	0.00973 (5.7%)	0.00785 (4.6%)	0.00714 (4.2%)
$\sigma \left(\left||\epsilon_{F_{X}}|\right|\right)$ [N]	0.315 (6.0%)	0.345 (6.6%)	0.310 (5.9%)	0.309 (5.9%)	0.314 (6.0%)
$\sigma \left(\left||\epsilon_{F_{Y}}|\right|\right)$ [N]	0.242 (4.6%)	0.392 (7.5%)	0.253 (4.8%)	0.218 (4.2%)	0.222 (4.2%)
$\sigma \left(\left||\epsilon_{L}|\right|\right)$ [M]	0.01551 (9.1%)	0.00681 (4.0%)	0.01040 (6.1%)	0.00567 (3.3%)	0.00588 (3.5%)

It can be generally seen that the model-based estimation methods provide an overall better performance compared to their polynomial-based segmentation counterparts. This is because they can more often correctly estimate the actual number of forces, in addition to having a lower mean error on the force locations. Note however, that the force magnitude errors are comparable for all methods. The computational times of the polynomial-based segmentation methods are significantly lower however, taking around 1–2 ms for completion. The UKF approach takes around 30 ms, while the least-squares optimization method takes over 8 s. This is the major drawback of the least-squares optimization method, making it unusable for practical real-time settings.

**FIGURE 5:**
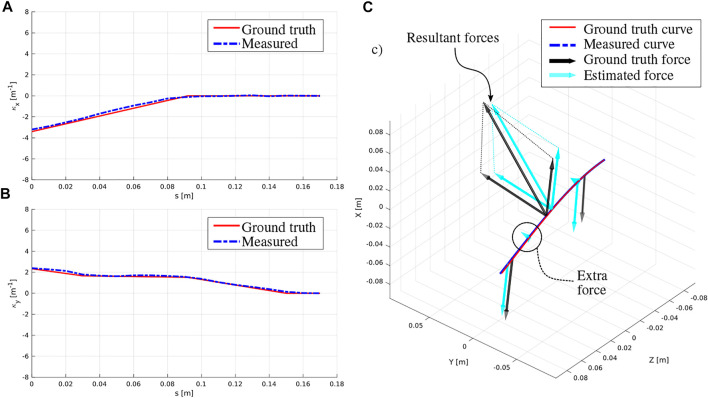
Example result for the case where three external point forces are applied onto the rod. **(A)** profile of the *x-*component of the curvature, **(B)** profile of the *y-*component of the curvature, **(C)** deformed rod showing a comparison between the ground truth and estimated shape and applied forces.

Figure 5 shows an example result for a configuration where three external point forces are applied onto the rod. The forces at the extremities are due to suspended weights, while the force in the middle is due to the ATI force sensor and the attached cable. The curvature profiles in the *x-* and *y-*directions clearly illustrate how the different point forces in given directions correspond to changes of slope. It can also be seen that the measured curvature, which is a linear interpolation of the discrete curvature measurements at each FBG location, deviates from the ground truth curvature. This can be due to a variety of reasons including: having a gap between the MCF and the inner lumen of the surrounding tube, friction between the MCF and the inner lumen of the surrounding tube which causes twist during bending, and FBG sensor accuracy itself. These directly impact the measured curvature *κ* and bend angle $\theta_{b}$ profiles, which are the key to accurate force estimation. Figure 5 also shows the deformed Nitinol rod and a comparison between the ground truth shape, estimated shape, and the applied forces. While the measured curvature may be noisy for direct force estimation, it can be readily employed to reconstruct the shape of the rod, with tip errors <2 mm. Furthermore, the close correspondence between the ground truth forces and locations with the estimated forces and locations can also be clearly seen. Notice that in this configuration, an extra force is estimated (see Figure 5). However, because its magnitude is very small compared to the other forces and less than $F_{min}$, it is assumed negligible and discarded. This is often the case since the model assumes four applied forces a priori, while in reality, only one to three forces are applied.

## 6 Distributed Forces

Thus far, the methods and experimental validations described concerned *point* force estimation. However, in some applications forces can be distributed. This section introduces some first works explaining how distributed forces can be estimated. Note that these methods concern distributed forces with constant magnitude.

### 6.1 Piecewise Polynomial-Based Segmentation of Curvature

It was previously pointed out that it is possible to estimate distributed forces using piecewise polynomial-based curvature segmentation. The method must be extended to allow for 1) second order polynomial detection, and 2) classification of polynomial segments to first or second degree. However, when curvature measurements are discrete, sparse, and require interpolation to provide extra smoothness (as is the case here since curvature measurements are only available at 10 mm intervals), it becomes very challenging to distinguish between first order and second order polynomials, especially if the curvature measurements are noisy as well. This task, however, can be simplified if extra information was given a priori such as the locations of the forces or the total number of applied forces. Since this information is not assumed available, we will not continue with this method for distributed forces estimation.

### 6.2 Model-Based Estimation

#### 6.2.1 Method Description

The model-based method is more general and flexible as the underlying Cosserat rod theory is general and can be used to model the rod’s forward kinematics using any type of external force. Hence, the strategy to estimate distributed forces is a simple expansion to the method developed for point forces in Section 3B. The difference is in the model’s input vector χ, which is now defined as:

$$\chi =\left[\begin{array}{c} f_{x1}\\ f_{y1}\\ L_{s1}\\ L_{e1}\\ f_{x2}\\ f_{y2}\\ L_{s2}\\ L_{e2}\\ \vdots \\ f_{xN}\\ f_{yN}\\ L_{sN}\\ L_{eN} \end{array}\right],$$ (16)

where $f_{xi}$ and $f_{yi}$ are the magnitudes of the x− and y− components of the $i^{th}$ external distributed force, respectively, and $L_{si}$ and $L_{ei}$ are the start and end locations of the distributed force along the arc length. The output curvature vector κ and the forward kinematics function g(χ) remain the same. The only difference is that distributed forces f were set to zero in the earlier implementation, whereas now they are not.

#### 6.2.2 UKF Simulation

**FIGURE 6:**
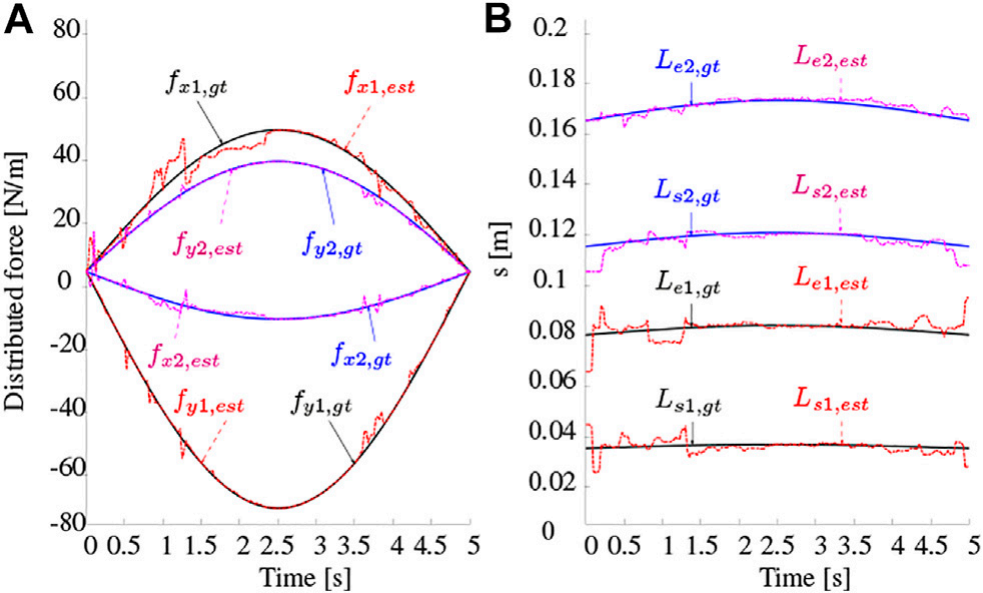
UKF simulation with two distributed forces applied onto a rod. The magnitudes and locations are varying sinusoidally over time (*gt* = ground truth, *est* = estimated).

To illustrate the feasibility of the proposed method, a simulation study is provided using the UKF approach. Note that the least-squares optimization approach can be employed in theory, but is disregarded in this study for its high computational cost. Here, two distributed forces with varying magnitudes and locations are applied onto the rod. The magnitudes and locations vary according to a sinusoidal profile as shown in Figure 6A. Gaussian noise with zero mean and 0.01 m^−1^ standard deviation was added to the curvature measurements. The UKF estimation of the distributed force magnitudes and locations is also shown for a simulation time of 5 s. Table 3 gives a summary on the absolute value of the errors between the ground truth and estimated forces and locations. It is clear that the UKF shows excellent performance in estimating both their magnitudes and locations. When comparing the mean magnitude and location errors with the experimental errors shown in Table 2, it is evident they are lower, and that the overall performance is better. Even though the results shown are for a simulation study only, they do give an indication that the performance in practice could be decent as well. Finally, a similar simulation was carried out by applying one point force and one distributed force. The same distributed force UKF model was able to estimate the point force magnitude and location to the same accuracy as outlined previously. This shows that the distributed force model is a generic model allowing for the estimation of generic force types.

**TABLE 3:** Simulation distributed force estimation errors over time.

	$f_{x}$ [N/m]	$f_{y}$ [N/m]	$L_{s}$ [m]	$L_{e}$ [m]
Mean	1.19 (1.6%)	0.47 (0.6%)	0.0018 (1.1%)	0.0017 (1.0%)
Max	10.21 (13.6%)	13.73 (18.3%)	0.0107 (6.3%)	0.0147 (8.6%)

## 7 Conclusion and Discussion

Estimation of forces along instrument bodies is a crucial aspect in medical navigation and handling. However, estimating such forces has proven to be challenging. Sensitive tactile sensors could solve this problem, but designing them to be robust and compact, featuring high spatial and force resolution has been found extremely difficult. The recent advent of compact MCFs opens up some opportunities for distributed contact force sensing, as demonstrated in this work. Here the instrument’s deformation itself can be exploited to provide estimates of the external forces. Two approaches have been developed to provide point and distributed force estimations using curvature information exclusively from a MCF. The resulting performance, for all methods, clearly indicates their feasibility for body force estimation. The different methods offer different advantages and result in an average of around 64.4% correct number of forces estimations, 5.5% force magnitude error, and 5.3% location error.

In the latter methods, the model-based UKF has shown to stand out when comparing accuracy, generalizability and computational time of the different methods. Furthemore, it must be noted that the Nitinol rod used in the validation experiments has a relatively high flexural rigidity, *EI* = 0.048 Nm^2^. In reality, medical instruments tend to have lower flexural rigidities. This is more beneficial as it allows for larger curvatures and an easier distinction between consecutive segments. It also overcomes minimum sensitivity issues regarding the MCF. Hence the proposed methods will work better for instruments with relatively low flexural rigidities. However, a key aspect to keep in mind is the elasticity of the instrument, which is necessary to ensure repeatability of the results.

Finally, a generalized method to estimate externally applied distributed forces was proposed, and illustrated that it can actually be applied for both point forces and distributed forces. Extensions to the proposed model can be easily implemented for diverse types of external loads. Furthermore, the feasibility of the proposed method was proved for forces with varying magnitudes and locations. Meaning that the method not only works for static loads, but also for quasi-static loads. Future work will focus on validating this method experimentally on elastic instruments with low flexural rigidities.

## Data Availability

The original contributions presented in the study are included in the article/Supplementary Material, further inquiries can be directed to the corresponding author.
